# Part B: Improvement of the Optical Properties of Cellulose Nanocrystals Reinforced Thermoplastic Starch Bio-Composite Films by Ex Situ Incorporation of Green Silver Nanoparticles from *Chaetomorpha linum*

**DOI:** 10.3390/polym15092148

**Published:** 2023-04-30

**Authors:** Nour Houda M’sakni, Taghreed Alsufyani

**Affiliations:** 1 Department of Chemistry, College of Science, Taif University, P.O. Box 11099, Taif 21944, Saudi Arabia; 2 Laboratory of Interfaces and Advanced Materials (LIMA), Faculty of Science, Monastir University, Monastir 5019, Tunisia

**Keywords:** green macroalga, red sea, *Chaetomorpha linum*, cellulose nanocrystals, starch, green silver nanoparticles, bio-nanocomposite films, optical properties

## Abstract

The study was used in the context of realigning novel low-cost materials for their better and improved optical properties. Emphasis was placed on the bio-nanocomposite approach for producing cellulose/starch/silver nanoparticle films. These polymeric films were produced using the solution casting technique followed by the thermal evaporation process. The structural model of the bio-composite films (CS:CL-CNC7:3–50%) was developed from our previous study. Subsequently, in order to improve the optical properties of bio-composite films, bio-nanocomposites were prepared by incorporating silver nanoparticles (AgNPs) ex situ at various concentrations (5–50% *w*/*w*). Characterization was conducted using UV-Visible (UV-Vis), Fourier Transform Infrared (FTIR), Scanning Electron Microscope (SEM) and Transmission Electron Microscope (TEM) to understand the structure–property relationships. The FTIR analysis indicated a reduction in the number of waves associated with the OH functional groups by adding AgNPs due to the formation of new hydrogen bonds between the bio-composite matrix and the CL-WE-AgNPs. Based on mathematical equations, the optical bandgap energy, the energy of Urbach, the edge of absorption (Ed), and the carbon clusters (N) were estimated for CS:CL-CNC and CS:CL-CNC-AgNPs (5–50%) nanocomposite films. Furthermore, the optical bandgap values were shifted to the lower photon energy from 3.12 to 2.58 eV by increasing the AgNPs content, which indicates the semi-conductor effect on the composite system. The decrease in Urbach’s energy is the result of a decrease in the disorder of the biopolymer matrix and/or attributed to an increase in crystalline size. In addition, the cluster carbon number increased from 121.56 to 177.75, respectively, from bio-composite to bio-nanocomposite with 50% AgNPs. This is due to the presence of a strong H-binding interaction between the bio-composite matrix and the AgNPs molecules. The results revealed that the incorporation of 20% AgNPs into the CS:CL-CNC7:3–50% bio-composite film could be the best candidate composition for all optical properties. It can be used for potential applications in the area of food packaging as well as successfully on opto-electronic devices.

## 1. Introduction

The development of the bio-composites was based on the environmental knowledge that was generated over time. The remaining natural starch is a vital biomaterial for making environmentally friendly materials. In order to improve the properties of these biomaterials, a new area was created, including the development of bio-nanocomposites [1]. This is a continuous polymer phase in which loads of at least one size on a nanoscale are dispersed. At this scale, the surface effects become predominant with volume effects in order to obtain original properties different from massive objects. The synthesized nanocomposites have demonstrated their versatility as a catalyst in the esterification response for the production of bio-diesel, a very good potential substitute for Gram-positive antimicrobial activity, as well as Gram-negative micro-organisms and an effective material for energy storage applications [2]. In this regard, nanostructures and nanocomposites, due to their small size, good catalytic activity, high surface area and outstanding selectivity, can play an important role in the near future [3]. Nanofillers, by their specific properties and the multitude of forms they can take, produce functional materials of interest in a number of areas such as electronics, medicine, cosmetics, optical physics and packaging [1]. 

Polymer matrices used for the production of CNC-reinforced composites could be divided into two parts: biodegradable and non-biodegradable polymers [4]. For example, natural polymers including by-products of cellulose, starch, natural rubber, and biopolymers such as polyhydroxyalkanoate (PHA), polylactic acid (PLA), polycaprolactone (PCL), etc., were widely used as biodegradable polymers to prepare bio-nanocomposites reinforced with cellulose nanocrystals [4,5]. Therefore, in our recent studies [6], we sought to reinforce the bio-composite film, with the introduction of a green *C. linum* silver nanoparticle in order to improve the optical properties of the biofilm. The study provided not only the optical property but also the characterization that led to the development of an optoelectronic bio-nanocomposite. 

Due to remarkable physical and chemical properties, noble metallic nanoparticles play their role in various domains such as biological markers [7], treatment of cancer tumors [8], fluorescence [9], improving the efficiency of solar panels [10] or the signal in Raman spectroscopy [11]. Moreover, through enhanced optical response, these nanoparticles are indirectly used to characterize the physical properties of other particles such as catalysis [12]. However, integrating metallic nanoparticles in the polymer matrix is a fundamental and critical industrial challenge. In fact, their load properties, their nature and their presence in the matrix considerably alter the mechanical, thermal, electrical or optical properties, as well as contributing to simplifying and reducing the cost of the transformed material. In recent years, silicon nanowires decorated with silver nanoparticles have also been identified as semi-conductor and antibacterial unidimensional synthetic nanomaterials [13]. AgNPs with diverse properties such as catalytic activity [14], Raman diffusion [15], good conductivity [16], anti-microbial [17] and optical activity [18] have generated considerable interest in the area of nanotechnology [19]. AgNPs can be synthesized using physical, biological and chemical approaches [20]. However, physical–chemical techniques are highly productive in designing well-defined nanoparticles, but they have certain limitations, such as the use of hazardous chemicals, high costs, time-consuming processes, and the generation of impurities [21]. Green synthesis has gained more interest and actively increased progress in the fields of science and industry due to its ecological, cost-effective and non-hazardous nature [22]. To synthesize AgNPs, a metal precursor, reducing agents, and a non-toxic stabilizing/capping agent were required [23,24]. However, the presence of biomolecules of natural active agents in plants plays a significant role in reducing and stabilizing nanoparticles [24,25]. The phytochemical substances involved in reducing and capping nanoparticles are terpenoids, flavonoids, phenols, alkaloids, polysaccharides, proteins, enzymes, amino acids, etc. [26]. In addition, other active agents were reported such as linalool, quinol, methyl chavicol, eugenol, chlorophyll, caffeine, theophylline, ascorbic acids, and so on [27,28]. Several methods have been developed for incorporating nanoparticles into a polymer matrix in two ways, which are known as ex situ and in situ [29].

Each organic semiconductor has its own energy levels that basically depend on the molecular structure. Thus, the electronic bandgap is the energy difference between the lowest unoccupied molecular orbital (LUMO) and highest occupied molecular orbital (HOMO) level [30]. This difference in energy reveals the bandgap that electrons can only penetrate by external excitation. In general, it is 1 to 3 eV for organic semiconductors and zero for conductors with overlapping valence and a conductive band. When it is high (>6 eV), the material is insulating, since it does not transfer electrons [31].

The objective of this study was to Integrate ex situ AgNPs as an electron donor reinforcement at the bandwidth of the bio-composite developed in our recent study [6] in order to develop new low-bandgap bio-nanocomposites for an optoelectronic application. The main requirement was to reduce the HOMO energy level as well as the bandgap of the polymers in order to increase the open circuit voltage of organic photovoltaic solar cells.

## 2. Experimental Work

### 2.1. Materials

Macroalgae thalli belonging to the order Cladophorales, *Chaetomorpha linum* were used as raw material to prepare AgNPs. Green algae were collected on the southwest coast of the Red Sea in Jeddah KSA (coordinates 21°37′41″ N and 39°6′11″ E). A CS:CL-CNC7:3–50% bio-composite (Table 1) was developed by reinforcing the thermoplastic starch matrix with cellulose nanocrystals (CL-CNCs) derived from *C. linum* algae biomass, according to the treatment detailed in our previous studies [6]. Silver nitrate-AgNO_3_ (99.8%) was purchased from VWR, PROLABO.

#### 2.1.1. Colloidal Silver Nanoparticle (AgNP) Synthesis

**Figure 1:**
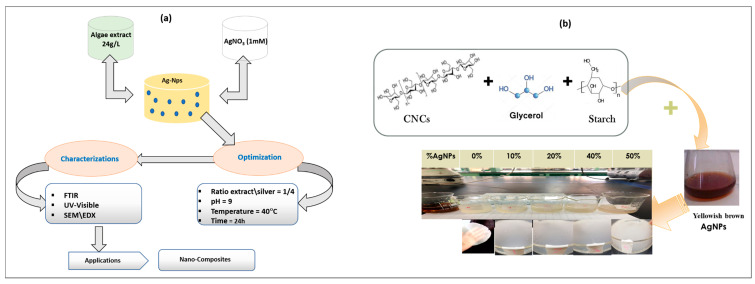
Schematic diagram of (**a**) elaboration of CL-WE-AgNPs from *C. linum*, and (**b**) elaboration of bio-nanocomposites.

The hydrosoluble polymers were obtained from 2 g of dry *C. linum* powder (CL-R) by extraction for 2 h in hot deionized water (1:40 w:w, 80 °C) with mechanical stirring. The mixture was filtered through a cloth and then centrifuged (5000 rpm) for 20 min to produce the CL-W fraction, as illustrated in Figure 1a. The filtrate was precipitated with the addition of 95% ethanol (40:60 v:v) to remove the remaining salts as well as low-molecular-mass polymers (proteins and polysaccharides) [32]. After decanting for 12 h (4 °C), the insoluble material was centrifuged out (5000 rpm) for 30 min to obtain the CL-WE fraction and recovered in deionized water. This fraction was used as a reducing and stabilizing agent for AgNPs synthesis [32]. To synthesize the colloidal AgNPs, 10 mL of seaweed extract was added to 90 mL of a 1 mM aqueous solution of silver nitrate (AgNO_3_) with constant stirring. The pH was adjusted to 9 by using 1 M NaOH solution to promote the reduction of Ag^+^ ions at ambient temperature. Within hours, the color changed from yellow to dark brown, which confirmed the formation of AgNPs [33]. The synthesized material was labeled CL-WE-AgNPs.

#### 2.1.2. Development of Optical Bio-Nanocomposite Films

To improve the performance of the bio-composite packaging, which was produced with the best formulation made for the biodegradable film (containing cellulose nanocrystals and thermoplastic starch), CL-WE-AgNPs were added in order to enhance the optical properties of bio-composite films.

Firstly, five solutions were prepared using the CS:CL-CNC7:3–50% bio-composite, and they were mixed successively with different compositions of CL-WE-AgNPs by weight with 190 mL of distilled water, stirred for five minutes and sonicated for 15 min. Then, the solutions were heated in a water bath at 85 °C for 30 min. The solution was moved into a Petri dish and stored in the oven for 24 h at 45 °C for drying. The films were peeled and retained in a desiccator (48 h) to control moisture (Figure 1b). The composition of the materials was fixed in cellulose nanocrystals, starch, and glycerol, which vary only with the percentage by weight of AgNPs, as shown in Table 1. Consequently, the percentage by weight of CL-WE-AgNPs was derived from the total quantity of mixture added. The resulting biofilms are named: CS:CL-CNC7:3-AgNPs (5%), CS:CL-CNC7:3-AgNPs (10%), CS:CL-CNC7:3-AgNPs (20%), CS:CL-CNC7:3-AgNPs (40%), and CS:CL-CNC7:3-AgNPs (50%) for the bio-nanocomposite films and CS: CL-CNC7:3–50% for the controlic bio-composite film.

### 2.2. Characterization Methods

#### 2.2.1. UV-Visible Analysis

For optical properties, the UV-Vis-NIR (JASCO; V670) (JASCO; V670, Easton, Portland, OR, USA) spectrophotometer was used to study the optical transmittance (T) and absorbance (A) of films prepared over the wavelength range of 190 to 900 nm (Figure 8a,b).

Determining the bandgap value of both amorphous materials and polymers is crucial for their applications. The most popular technique to stimulate the bandgap is a measurement of the optical absorption coefficient. This coefficient was determined by mean absorbance (A), and Equation (1) was followed [34]:

(1) $$\alpha \;=\frac{2.303\;A}{d\left(\mathrm{cm}\right)}$$

where d = 0.02 cm, and the film thickness was determined by a Vernier caliper. The functions of the absorption coefficient with incident photon energy for bio-composite films CS:CL-CNC7:3–50% and for bio-nanocomposite films CS:CL-CNC7:3-AgNP (5–50%) are shown, respectively, in Figure 9a,b.

The energy bandgap (Eg) deviation of all the prepared films was determined by intercepting the plotted linear portion (α hν)^2^ versus hν, as shown in Figure 10a, pursuing Tauc’s method (Equation (2)) [35,36]:

(2) $$\alpha h\nu \;=B{\left(h\nu \;-\mathrm{Eg}\right)}^{n}$$

where B is the width parameter of the absorption edge, hν is the incident photon energy calculated from hν (eV) = 1240/λ (nm), and n is the factor that takes 3/2 or ½ for direct transitions and 2 or 3 for indirect transitions relaying on the forbidden or allowed transition, respectively.

For determining the band tail that refers to the width of localized states, the absorption coefficient α(v) near the band edge as exponential dependence of photon energy (hv) was determined from the Urbach relationship (Equation (3)) [37,38]:

(3) $$\alpha \;\left(h\nu \right)=\alpha_{0\;}e^{\left(\frac{h\nu }{E_{e}}\right)}$$

where $\alpha_{0}$ is known as the constant and Ee denotes the band tail (Urbach tail), referring to the localized state’s width (Figure 10b).

The number of carbon atoms (N) in a cluster is calculated from the optical energy bandgap (Eg) using the following relation (Equation (4)) [39,40]:

(4) $$\mathrm{Eg}=34.4/\sqrt{N}$$

#### 2.2.2. FTIR Analysis

Several stages involved in the development of bio-nanocomposite films were studied by FTIR (Thermo spectrophotometer, Nicolet IR 200, Madison, WI, USA). FTIR spectra were recorded between 4000 and 400 cm^−1^ and compared with data already reported to distinguish the signal in a specific manner.

#### 2.2.3. SEM and TEM Analyses

The JEOL model JEM-2000FX (Tokyo, Japan) instrument operated at an accelerating voltage of 200 Kilo voltage used to determine SEM (scanning electron microscope), EDX (energy-dispersive X-ray spectroscopy), and TEM (transmissions electron microscopy) measurement.

SEM and EDX images were taken for the characterization of the morphology, and the microstructures of all the materials were obtained at different stages of the bio-nanocomposite film development process.

In order to better clarify the morphology and size of the particles, TEM analysis was applied. A few drops of sonicated powdered sample were prepared and placed on a carbon-coated copper grid and air-dried for 1 h. The CL-WE-AgNP sample was selected for TEM analysis. 

## 3. Results and Discussion

### 3.1. Impact of AgNPs Density on Optical Responses of Bio-Nanocomposite Films

#### 3.1.1. Synthesis of Colloid Silver Nanoparticles (AgNPs)

AgNPs (5, 10, 20, 40 and, 50%) were ex situ incorporated in (CS:CL-CNC7:3–50%) biofilm employing the matrix containing CL-CNC from *C. linum* (3 g), CS (7 g) and 50% glycerol as plasticizer agent to improve the optical properties of our previous biofilm [6]. The reduction of AgNPs was carried out by UV-Visible, SEM-EDX, TEM, and FTIR.

**Figure 2:**
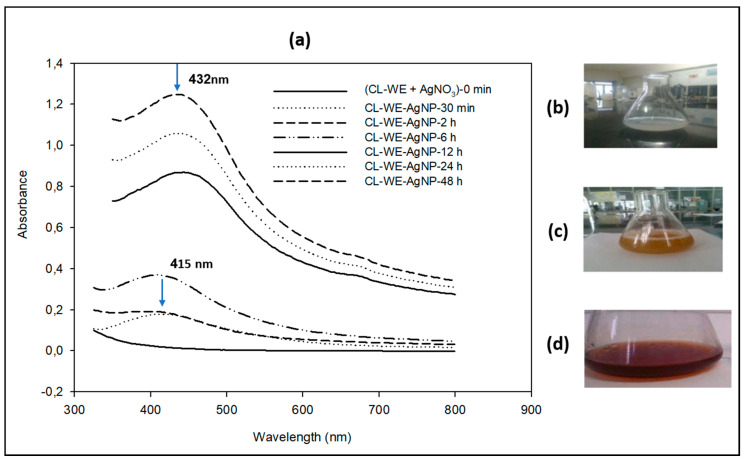
(**a**) UV-Vis absorption spectrum of silver nanoparticles CL-WE-AgNPs synthesized using aqueous dialyzed extract of fresh *C. linum* (CL-WE) as a function of reaction time, (**b**) beginning of the reaction (pale white–yellow), (**c**) after 30 mn (yellow–brown color) and (**d**) after 48 h (brownish color) at 40 °C, and pH = 9.

The reduction of the Ag^+^ ion into AgNPs was analyzed by color change (Figure 2a–d). Before adding the solution (AgNO_3_, 1 mM), the supernatant of the *C. linum* extract precipitated in ethanol (CL-WE) was pale white–yellow (Figure 2b); it turned to a yellow–brown color after 30 min of contact (Figure 2c) and then brownish at the end of the reaction with the ions (after 48 h of contact) (Figure 2d).

These results were confirmed by UV-Visible characterization spectrophotometry, which is a technique that proved to be very useful for the rapid analysis of colloidal solutions of AgNPs. This helps to determine whether the synthesis process was terminated by the formation of nanoparticles. The formation and stability of the reduced silver nanoparticles in the colloidal solution were monitored by UV-Vis spectrophotometric analysis [41]. The UV-Vis spectra showed a maximum absorbance at 415 nm that increased with the incubation time of silver nitrate with *C. linum* extract (Figure 2a) [42]. The presence of an absorbance peak at approximately 415 nm makes clear the formation of AgNPs in the solution, which is due to surface plasmon resonance (SPR) electrons present on the nanoparticle surface. The intensity of the SPR band increased with reaction time (30 min, 2 h, 6 h, 12 h, 24 and 48 h) and showed a maximum absorbance at 432 nm after 48 h (Figure 2a), indicating the synthesis of the AgNPs [42]. It is reported earlier that the absorbance at about 430 nm for silver is a feature of these noble metallic particles [43,44]. These results confirmed that 48 h time is the longest synthesis time at the present temperature and pH. It was observed that with an increase in the contact time between CL-WE and AgNO_3_, the absorption peak shifted to a higher wavelength (from 415 to 432 nm), indicating an increase in the size of the AgNPs synthesized extract. These results are similar to those reported in the literature [41,42,45].

#### 3.1.2. Morphological Analysis of Synthesized AgNP and Its By-Products

**Figure 3:**
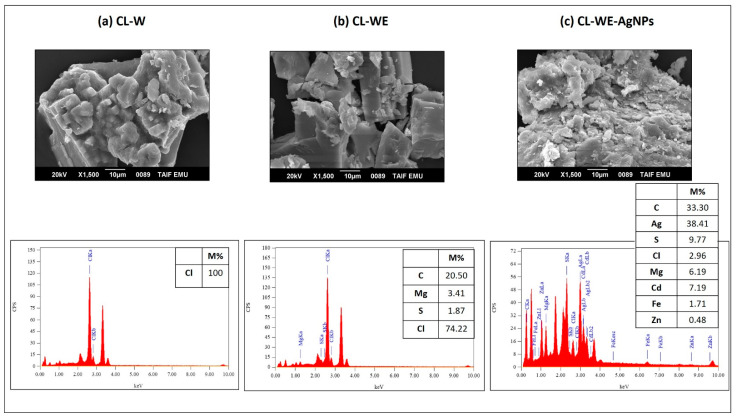
SEM and EDX image of silver nanoparticles synthesized steps: (**a**) CL-W, (**b**) CL-WE, (**c**) CL-WE-AgNPs.

A scanning electron microscope with an energy-dispersive X-ray spectrometer (SEM-EDX) was used to determine the silver concentration of the nanoparticles (Figure 3a–c). However, the EDX analysis of the aqueous extract of *C. linum* (CL-W) (Figure 3a) showed a high percentage of chlorine and salt, while after precipitation with ethanol (Figure 3b), the percentage of chlorine was decreased, so we note the presence of sulfur specific for glucosamines, which confirms the role of ethanol purification. After the reduction of the silver ions with the aqueous extract (CL-WE) (Figure 3c) which performs both reducing and stabilizing effects, a new distinct peak was detected at 2.983 keV in the CL-WE-AgNPs. Prasad, Kambala [46] have shown that AgNPs generally exhibit an absorption peak in the region of 3 keV due to the phenomenon of surface plasma resonance. The appearance of this peak (Figure 3c) confirmed the presence of elemental silver in the nanoparticles thus produced (CL-WE-AgNPs) with a silver concentration of around 38.41 ± 1.06%, which was detected after an incubation of 48 h. The morphology of the CL-WE-AgNPs shows that several co-components appear alongside silver: in particular, iron, magnesium, zinc, cadmium, chlorine, sulfate… This is due to the reduction of silver by the aqueous *C. linum* extract, which was accompanied by the reduction of those trace metals and led to the formation of co-nanoparticles as a trace. Despite the low metal content (zinc and cadmium ≤ 1 ppm, iron ≤ 2 ppm, and magnesium ≤ 5 ppm per mass) that coexist with silver (originally VWR Chemicals commercial silver nitrate), ethanol precipitates of aqueous extract have been able to reduce these metals and obtain co-nanocomposites alongside nanocomposite silver synthesis. This explains the effect of ethanol precipitation of the plant extract that characterizes our method of green synthesis of AgNPs, which promotes the increase in the reducing properties of the plant extract following the purification of ethanol.

**Figure 4:**
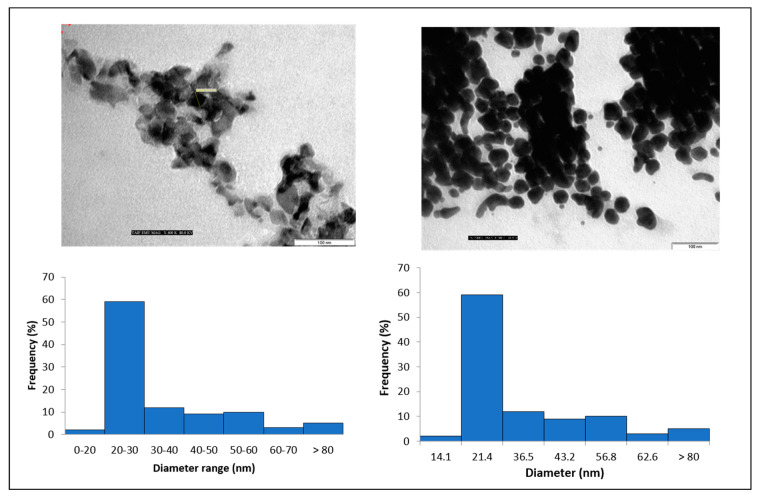
TEM image of CL-WE-AgNPs (48 h).

Figure 4 represents the TEM image of CL-WE-AgNPs (48 h) determining that the AgNPs are well defined and are spherical with slight agglomeration. The presence of agglomeration could be due to the drying effects produced during sample preparation [47,48]. The aggregation behavior of AgNPs is mostly affected by pH and electrolyte concentration [49], while the presence of biomolecules can improve particle stability due to the biomolecular coronary effect [49]. In our work, we show that a degree of detected aggregation can be attributed to the ethanol precipitation effect of water extract (CL-W) that removes the salt that coexists with biomolecules. As shown, the size of nanoparticles increases with increasing concentration of AgNO_3_. The Ag size range thus detected varied from 20 to 30 nm with an average of 21.4 nm. In comparison to the previous research, it was concluded that AgNPs with 20 nm size have a plasmon resonance band around 430 nm (violet absorption) and are brown [50].

#### 3.1.3. FTIR Analysis of Synthesized AgNPs and Its By-Products

**Figure 5:**
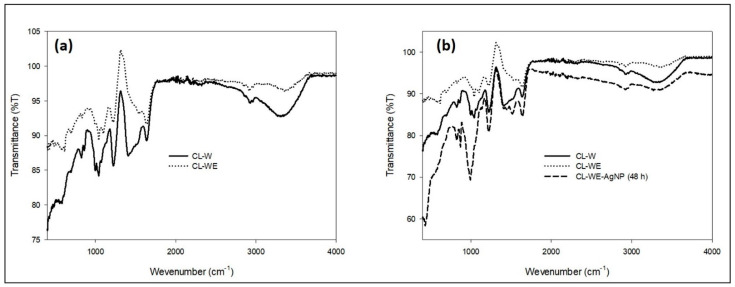
FTIR analysis of (**a**) *C. linum* water extract before and after purification with ethanol precipitation and (**b**) colloidal silver nanoparticle CL-WE-AgNPs synthesis.

The biomolecules present in the root extract of *C. linum* played an active role in reducing Ag^+^ to AgNPs, as confirmed by the FTIR analysis. Figure 5a,b represent the FTIR spectrum of *C. linum* extract before and after ethanol precipitation. The FTIR spectra of dried *C. linum* extract before purification by ethanol (CL-W) (Figure 5a) have shown many peaks at 614 cm^−1^, 645 cm^−1^, 706 cm^−1^, 839 cm^−1^, 863 cm^−1^, 924 cm^−1^, 1004 cm^−1^, 1047 cm^−1^, 1095 cm^−1^, 1224 cm^−1^, 1412 cm^−1^, 1538 cm^−1^, 1645 cm^−1^, 2297 cm^−1^, 2842 cm^−1^, 2910 cm^−1^, 2938 cm^−1^, 3279 cm^−1^ and 3338 cm^−1^. However, the FTIR spectra of dried *C. linum* extract after purification with ethanol precipitation (CL-WE) have shown disparate peaks at 614 cm^−1^, 645 cm^−1^, 924 cm^−1^, and 2297 cm^−1^ specifying aliphatic bromo compound, aliphatic-chloro compound, nucleic acid (other phosphate-containing compounds) [51,52] and nitrile compounds [53], respectively. Meanwhile, the decrease in the peak at 1539 cm^−1^ is of amide II [54], explaining the effect of precipitation in ethanol which eliminates the salt compounds and minimizes the presence of free protein and increases the level of polysaccharides in the solution [32]. The appearance of the strong peak at 1220 cm^−1^ is specific to the asymmetric stretching vibration of sulfate groups commonly available in *C. linum* in the form of sulfated polysaccharides [55], which are used for the stabilization of AgNPs [56]. However, the corresponding bands observed at 1545 cm^−1^ and 1643 cm^−1^ are assigned successively to the amide II from proteins and the stretching vibration of the (NH) C=O group. After the reduction of AgNO_3_, the shift of the bands from 1538 cm^−1^ and 1524 cm^−1^ is attributed to the involvement of the secondary amines in the reduction process and the binding of the (NH) C=O group with nanoparticles. Since a member of the (NH) C=O group within the cage of cyclic peptides is involved in stabilizing the nanoparticles, the shift of the (NH) C=O band is quite small. Thus, the peptides play a major role for the reduction of Ag^+^ to AgNPs. New bands in CL-WE-AgNPs at 1133 cm^−1^ and 1467 cm^−1^ (Figure 5b) may be attributed as vibrations of the glycosidic C-O bond (C-O-C) stretches from carbohydrates as well as to C=C-C, aromatic ring stretch, and the aromatic compound.

### 3.2. Characterization of Bio-Nanocomposite Films

#### 3.2.1. Morphological Analysis

**Figure 6:**
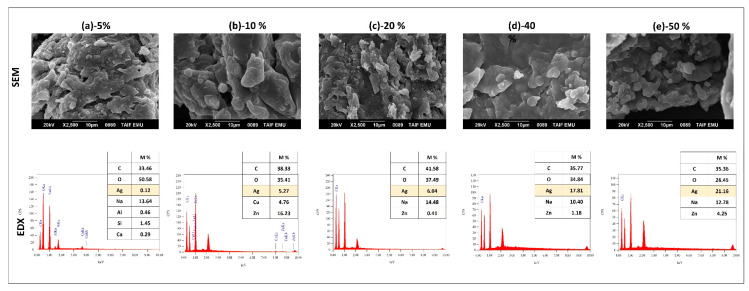
SEM and EDX analysis of bio-nanocomposite films CS:CL-CNC7:3-AgNPs (5–50%).

To analyze the surface morphology of the films produced, and to show the distribution of AgNPs in the bio-nanocomposites (Starch/Cellulose/AgNPs) thus formed, a scanning electron microscope with an energy-dispersive X-ray spectrometer (SEM-EDX) was used. Figure 6a–e provides specific information about the structure and changes in the optical properties of films resulting from the addition of AgNPs. The SEM image of the film without AgNPs (CS:CL-CNC7:3–50%) in one of our recent studies [6] shows a porous surface with rough tufts. In contrast, the SEM images of the films with AgNPs (CS:CL-CNC7:3-AgNP5–50%) (Figure 6a–e) show more or less smooth surfaces with the emergence of some small aggregates corresponding to CL-WE-AgNPs in the form of white markings merged into nanoclusters [57,58]. The SEM images (Figure 6c) show a better distribution of CL-WE-AgNPs in CS/CL-CNC7:3–50% films with a percentage of 20% of AgNPs, indicating sufficient interfacial interaction with the matrix of the CS/CL-CNC mixture and the CL-WE-AgNPs [59].

#### 3.2.2. FTIR Analysis

**Figure 7:**
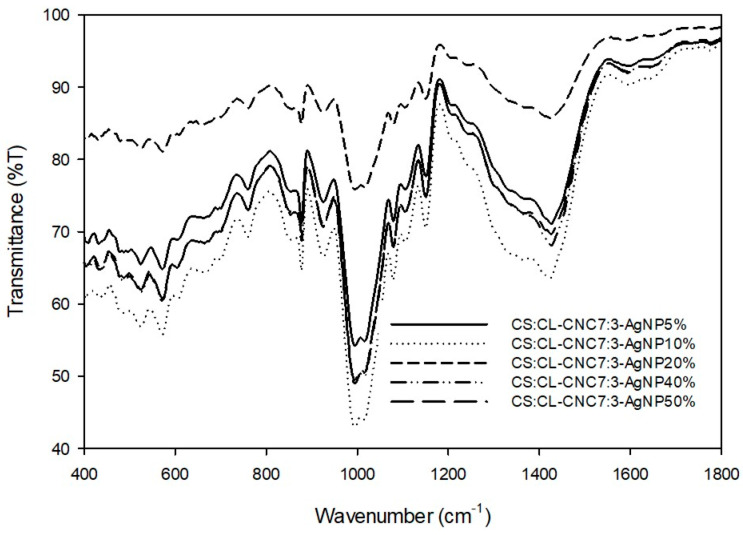
FTIR analysis of development bio-nanocomposite films (400–1800 cm^−1^).

After the preparation of the bio-nanocomposites by the modification of CS:CL-CNC7:3–50% films, by the incorporation of different levels of CL-WE-AgNPs (5–50%), FTIR measurements (Figure 7, Table S1) were performed to verify the formation of chemical bonds between the functionalized CL-WE-AgNPs and the matrix of CS:CL-CNC7:3–50%, which was carried on from our previous article for the current study [6]. The shape of the curves showed that the interactions of the matrix chains increase with the velocity of the nanoparticles (Figure 7). For all the matrices (CS:CL-CNC7:3–50%-AgNPs), new bands have appeared (1642 cm^−1^ and 1787 cm^−1^) showing a better interaction in the order of CL-WE-AgNPs rate: 5%, 10%, 40%, 50%, to 20%. Although the bio-nanocomposite containing 20% CL-WE-AgNPs seems best in terms of composition, it suggests a good intermolecular interaction between the different compositions of the matrix.

The band appeared at 1643 cm^−1^ specifying the groups of water released from CL-CNC, as we stated in one of our recent studies [6]. The absorbance increases as CL-WE-AgNPs are added, indicating that water absorption is inversely proportional to the proportion of hydrophilic CL-WE-AgNPs [60,61,62]. However, the band around 1787 cm^−1^ is attributed to the elongational vibrations of the C=O bond which occur due to the presence of hemicellulose residues from the CL-WE-AgNPs matrix on cellulose chains [60,63].

The number of waves associated with OH functional groups at 3321 cm^−1^ was reduced due to the formation of new hydrogen bonds between the bio-composite matrix and the CL-WE-AgNPs [64]. A similar result was reported by previous research, which is explained in terms of weakened hydrogen bonding due to electron delocalization [65,66].

### 3.3. Optical Properties of the Synthesized Bio-Nanocomposites Films: The Ability to Protect Films against UV

**Figure 8:**
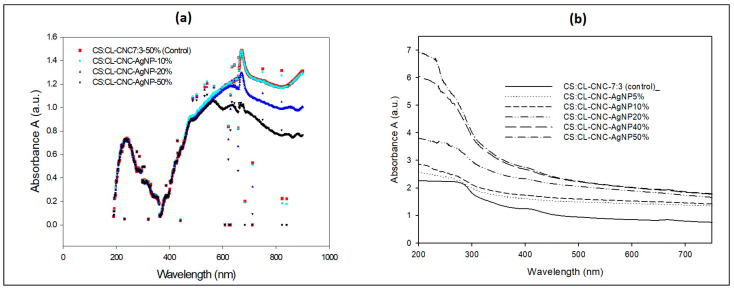
(**a**) UV-visible absorption spectra of bio-composite formulation with the integration of CL-WE-AgNPs (10%, 20%, and 50%), and (**b**) Optical absorbance spectra of development bio-nanocomposite films.

The absorbance of the bio-composite films of CS:CL-CNC:7:3–50% (control) [6] and with 5 to 50% of CL-WE-AgNPs was incorporated over the wavelength range of 190 to 900 nm, as shown in Figure 8a. As we stated in one of our recent studies [6], the starch/cellulose/glycerol-based bio-composite film exhibited UV absorbance at 273 nm but was not present in visible regions. In contrast, CS-CL-CNC-AgNPs (5–50%) films showed strong absorbance bands in the UV range around 212–300 nm (Figure 8a), which was due to the presence of AgNPs in the matrix. This explains that these bio-nanocomposites could be used in different fields of application. This is due to efficient UV absorbers, mainly for UV-C rays (100–280 nm), but also for UV-B rays (280–315 nm). He also noted that the intensity of the absorption peaks increases with the increase in the AgNPs content in CS-CL-CNC-AgNPs films (5–50% by weight), explaining a higher UV absorption ability for CS-CL-CNC-AgNPs 50% film compared to other matrices. Figure 8b revealed a strong absorption peak between 336 and 600 nm with a maximum absorbance of about 420–434 nm, which is the typical plasmon resonance band of AgNPs [33,67]. The peak indicates that the Ag^+^ ions in the solution (AgNO_3_, 1 mM) were successfully reduced to AgNPs.

**Figure 9:**
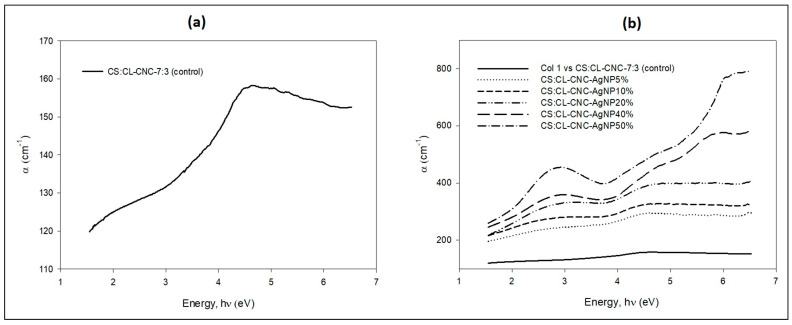
Optical absorption coefficient as a function of photon energy of (**a**) CS-CL-CNC7:3–50% bio-composite film [6] and (**b**) CS-CL-CNC-AgNPs (5–50%) development bio-nanocomposite films at ambient temperature.

The absorption coefficient (α) of the bioplastic film (CS-CL-CNC7:3–50%) is calculated according to Equation (1) and illustrated in (Figure 9a,b). The α-values of the film (Figure 9a) are high and draw a broad spectrum in the spectral region > 1.55 eV, which recommends the film for specific applications in the field of solar cells. Two absorption peaks also appeared at hν ≈ 2.1 eV and 4.6 eV in the visible and UV spectra range, respectively. These peaks are attributed to the π–π* transition between bonding and molecular orbital antibonding [68,69].

The band structure of solid materials can be identified by studying the optical absorption spectrum [70,71]. Optical absorption studies on polymer blend films without and containing various concentrations of CL-WE-AgNPs (5–50%) are presented in Figure 9b. It was carried out to determine the optical constants: namely the position of the edge of the fundamental band and the optical bandgap (Table 2).

Figure 9b demonstrated the optical absorption coefficients versus the photon energy of the biofilm mixture of CL-NCN and starch (CS-CL-CNC7:3–50%) and the bio-nanocomposites films (CS-CL-CNC-AgNPs (5–50%). In particular, the optical absorption coefficients of the CS-CL-CNC7:3–50% film decrease as the AgNP content increases (Table 2). This reduction in Ed is attributed to the increase in the number of charge carriers by the structural modifications of the polymer matrix, which is due to molecular interactions between polymeric chains and CL-WE-AgNP [72]. They are also related to the changes in the number of electrons and the holes in the conductive and valence bands [73]. Specifically, the optical absorption edge shift explains the electronic coupling between CL-WE-AgNPs and CS-CL-CNC7:3–50% [72,73].

**Figure 10:**
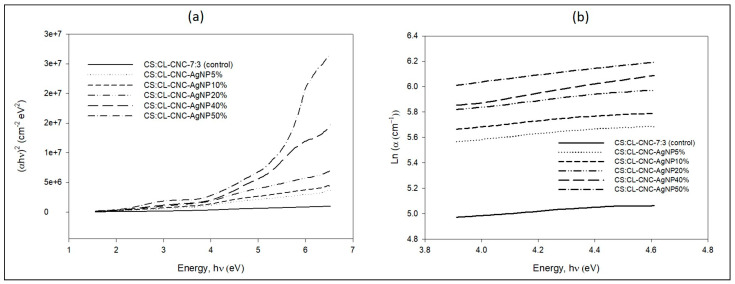
(**a**) Relationship between (αhν)^2^ verses photon energy (hν), (**b**) Absorption coefficient Ln α versus photon energy for CS-CL-CNC7:3–50% bio-composite film [6], and CS-CL-CNC-AgNPs (5–50%) development bio-nanocomposite films at ambient temperature.

The optical bandgap energy (Eg) is the most interesting parameter for integrated optical optoelectronic and photovoltaic devices [74]. Therefore, by extrapolating the linear region to the abscissa, we obtain the energy of the optical bandgap of the amorphous material (Figure 10a). Table 2 shows that the energy value of the bandgap of the CS-CL-CNC7:3–50% film without CL-WE-AgNPs is 3.12 eV and decreases with increasing AgNPs content to 2.58 eV for the CS:CL-CNC-AgNPs 50% nanocomposite film. In this case, the bandgap energy is lower (<3 eV), indicating that it is a semi-conductor [75]. In particular, the reduction of Eg of the polymer matrix of the different films is attributed to the fact that the content of CL-WE-AgNPs is responsible for modifying the degree of disorder of the polymer as well as its structure. As a result, these changes reflect the localized states in the bandgap, which are responsible for decreasing the bandgap energy of the polymer [39,76]. Thus, the reduction in the bandgap is due to the addition of CL-WE-AgNPs in the CS:CL-CNC-7:3–50% matrix, which was carried on from our previous article for the current study [6]. This is due to the increased carrier–carrier interaction in the valence and conduction bands and subsequently the displacement of the valence and conduction band. However, the decrease in Eg reflects the formation of charge transfer complexes (CTCs) as trap levels between the bands of the HOMO, which is mainly carried by the silver metal and is an MO type Ag-d(π), and the LUMO, which is carried by the polymer matrix and it is an MO type (π*) [77]. This highlights the good miscibility between the CL-WE-AgNPs and the polymer matrix [39].

The Urbach curve is shown in Figure 10b, and the logarithm of the absorption coefficient (α) is plotted as a function of the photon energy (hv). Table 2 groups the estimated values of the tail of the samples (Ee) which were obtained by the inverse of the slope of the linear part of these curves (Equation (3)). The absorption edge called the Urbach energy, and it depends on induced disorder, static disorder, temperature, thermal vibrations in the lattice, strong ionic bonds and on average photon energies [78]. In our study, the temperature and the thickness of films were fixed, respectively, at ambient temperature and 0.02 cm. It is apparent in Figure 10b that the incorporation of CL-WE-AgNPs significantly reduces the absorption edge and decreases the Ee values in the films (Table 2) [79]. Indeed, the values of Ee decrease from 6.89 to 3.62 eV with the increase in the concentration of CL-WE-AgNPs from 0 to 50% by weight. This decrease in the Urbach energy is the result of the decreased disorder of the biopolymers matrix, and/or this was attributed to an increase in crystalline size [78]. Results were explained by several studies, showing that AgNPs [39] and other nanoparticles such as carbon nanotubes [79] lead to a redistribution of states from the band to the tail and thus promote a large number of tail-to-tail transitions [39,79]. Other studies showed that the width of the Urbach tail decreased by moving from thicker film to finer film, which meant from order to disorder [80]. However, the addition of CL-WE-AgNPs costs a charge transfer complex and reduces both Eg and Ee. At the same time, nanocomposite films achieve a good transparency of about 20%, and these values are suitable for certain applications. The correlation between Urbach energy and film thickness should be investigated.

The carbon atom number in clusters of CS-CL-CNC7:3–50% films and different bio-nanocomposite films were calculated (Equation (4)) and are regrouped in Table 2. The value of N for a bio-composite film without CL-WE-AgNPs is around 121.56, which increases to 177.78 in CS:CL-CNC-AgNPs 50% bio-nanocomposites. The increase in N value is due to the conjugation in monomer units of CS:CL-CNC7:3–50% matrix post embedding of AgNPs. Meanwhile, the band tail and the number of carbon clusters in the samples increased with increasing AgNPs contents [39].

The increase might be due to the breaking of electrons in the C–H bonds due to the liberation of hydrogen [81]. During irradiations, there is the release of gases from the polymeric material. These released gases such as H_2_, H, CO, and CO_2_ can be led to the carbonaceous clusters (being rich charge carriers) in the polymer matrix. This carbonaceous cluster impacts the various physical properties of the polymeric material. Hence, the increase may be due to the carbon network and bonding of polymer–metal, and it ensures the conductivity of polymers [40]. Another study also shows that the value of the optical bandgap Eg shows a decreasing trend with the fluence of the irradiated ions and with two kinds of ions (Si^8+^ and Ne^6+^) [82]. In addition, the number of carbon atoms per conjugation length increases with the increase in the ion dose built into the bio-composite matrix [82]. The formation of these clusters in polymer films with ion irradiation has been investigated extensively [82,83], and it is explained by the carbon clusters, which are supposed to be carriers in electrical conductivity, being formed along the latent pathways of energy ions in polymers.

## 4. Conclusions

The improvement of the optical properties of the bio-composite film was obtained by integrating ex situ variable percentages of CL-WE-AgNPs (5–50%) synthesized from *C. linum* by green means. SEM/EDX confirmed a uniform dispersion of CL-WE-AgNPs in the bio-composite matrix with small agglomerations. A fundamental study of the optical properties of films was carried out to determine the absorption of UV with the presence of AgNPs in composites. However, a decrease in optical bandgap, edge absorption, and Urbach energy was observed compared to the CS:CL-CNC7:3–50% film developed in our recent study. The bandgap of the bio-composites decreases from 3.12 to 2.58 eV with the increase in the AgNPs content to 50%. In this case, the bandgap energy is lower (<3 eV), indicating that it is an improved semi-conductor. The decrease in Urbach energy results from a decrease in the matrix of biopolymers and/or an increase in crystalline size. Furthermore, the cluster carbon number increased, respectively, from 121.56 to 177.75 from bio-composite to bio-nanocomposite with 50% AgNPs. This is due to the presence of a strong H-binding interaction between the bio-composite matrix and the AgNP molecules. Consequently, the incorporation of AgNPs into the bio-composite film CS/CNC costs a load transfer complex and reduces Eg and Ee. At the same time, nanocomposite films attain a good transparency of about 20%. It could be applied to potential applications in the field of food packaging and can be used successfully on opto-electronic devices. Due to the promising properties of bio-composites, several new ones are emerging, and the assessment of their risks requires an individual approach of each nanomaterial. As a result of concerns about the safe use of bio-nanocomposites, further research into their mechanical properties and biological activity is required to provide a clear response regarding how and what nanomaterials can be a viable alternative to applications in many different areas.

## Figures and Tables

**Table 1:** Composition of the cellulose nanocrystals, starch, glycerol, and CL-WE-AgNPs used in the investigation for the development of bio-composite and, bio-nanocomposites films.

Sample Code	CL-CNC (g)	% NaOH (mL)	CS (g)	Distilled Water (mL)	Glycerol (mL)	CL-WE-AgNPs (mL)
CS:CL-CNC7: 3–50% [6]	3.00	40	7.00	140	90.0	-
CS:CL-CNC7:3-AgNPs 5%	2.85	38	6.65	133	85.5	9
CS:CL-CNC7:3-AgNPs 10%,	2.70	36	6.30	126	81.0	18
CS:CL-CNC7:3-AgNPs 15%	2.55	34	5.95	119	76.5	27
CS:CL-CNC7:3-AgNPs 20%	2.40	32	5.60	112	72.0	36
CS:CL-CNC7:3-AgNPs 40%	1.80	24	4.20	84	54	72
CS:CL-CNC7:3-AgNPs 50%	1.50	20	3.50	70	45	90

**Table 2:** Values of absorption edge (Ed), bandgap (Eg), band tail (Ee), and carbon cluster number N of CS-CL-CNC7:3–50% bio-composite film [6], and CS-CL-CNC-AgNPs (5–50%) development bio-nanocomposite films, at ambient temperature.

	Absorption Edge (Ed) (eV)	Optical Bandgap (Eg) (eV)	Urbach Energy (Ee) (eV)	Carbon Cluster Number (N)
**CS:CL-CNC7:3–50%** [6]	2.47	3.12	6.89	121.56
**CS:CL-CNC-AgNPs 5%**	1.51	2.91	5.41	139.74
**CS:CL-CNC-AgNPs 10%**	1.44	2.88	5.21	142.67
**CS:CL-CNC-AgNPs 20%**	1.32	2.82	4.25	148.80
**CS:CL-CNC-AgNPs 40%**	1.20	2.76	3.65	155.35
**CS:CL-CNC-AgNPs 50%**	1.08	2.58	3.62	177.78

## Data Availability

The data presented in this study are available on request from the corresponding author.

## Supplementary Materials

The following supporting information can be downloaded at: https://www.mdpi.com/article/10.3390/polym15092148/s1, Table S1: Assignment of bands found in FTIR spectra of different isolated water extract, silver nanoparticles and bio-nanocomposite.
