# Supplementary material for: Part B: Improvement of the Optical Properties of Cellulose Nanocrystals Reinforced Thermoplastic Starch Bio-Composite Films by Ex Situ Incorporation of Green Silver Nanoparticles from Chaetomorpha linum
Source: Polymers (Basel). 2023 Apr 30;15(9):2148. doi: 10.3390/polym15092148 (PMC10180543; doi:10.3390/polym15092148)
Supplement: Supplementary file 1 [file polymers-15-02148-s001.zip › polymers-2335655-supplementary.pdf]

Supplementary Information

Part B: Improvement of the Optical Properties of Cellulose Nanocrystals Reinforced Thermoplastic Starch Bio-Composite Films by Ex Situ Incorporation of Green Silver Nanoparticles from *Chaetomorpha linum*

Nour Houda M'sakni [1,2,*,†] and Taghreed Alsufyani [1,*,†]

[1] Department of Chemistry, College of Science, Taif University, P.O. Box 11099, Taif 21944, Saudi Arabia
[2] Laboratory of Interfaces and Advanced Materials (LIMA), Faculty of science, Monastir University, Monastir 5019, Tunisia
* Correspondence: nour.h@tu.edu.sa (N.H.M.); taghreed.alsufyani@tu.edu.sa (T.A.)
† These authors contributed equally to this work.

Supplementary Tables

Supplementary Table 1. Assignment of bands found in FTIR spectra of different isolated water extract, silver nanoparticles and bio-nanocomposite

	**Main peak (cm^{-1}) from isolated water extract, silver nanoparticles and bio-nanocomposite**							
	**CL-W**		**CL-WE**		**CL-WE-AgNp**		**CS-CL-CNC-AgNP 20%**	
	Peak (cm^{-1})	% T	Peak (cm^{-1})	% T	Peak (cm^{-1})	% T	Peak (cm^{-1})	% T
O-H stretching from water molecules	3338.27	96.45	3313.79	96.14	3350.51	91.05	3321	91.92
	3279.10	96.56	3275.01	97.12	3279.10	90.88	-	-
C-H stretching Lipid – carbohydrate Mainly vas) stretching$_3$(CH$_2$) and *vs.* (CH	2938.33<br>2909.76	96.42<br>94.75	2948.8<br>2913.00	96.42<br>96.95	-<br>2922.00	-<br>90.80	2942	93.30
	2842.43	95.70	2862.83	96.86	2870.99	91.69	2709<br>2532	93.70<br>94.10
stretching vibrations of the C=N chain of Nitrile compounds [53] (Subrhamanian, Suriyamoorthy, & Rajasekar, 2017)	2297	97.04	-	-	-	-	-	-
	-	-	-	-	-	-	1787	96.88
	-	-	-	-	-		1642	98.60
Amide I from proteins Stretching vibration of the (NH) C=O group	1644.7	89.27	1644.60	91.86	1640.61	84.37	1608	96.76
Amide II from proteins	1538.54	88.65	1544.66	93.11	1524.26	85.09	1597	96.38
C=C-C, Aromatic ring stretch, Aromatic compound	-	-	-	-	1467.12	86.06	1434	85.76
O-H bend, Alcoholic group	1412.03	87.04	1414.07	96.14	1416.11	86.42	1348	85.60
Asymmetric stretching vibration of sulfate groups	1224.30	85.43	1220.22	91.58	1218.18	80.61	1218	92.62
Stretching vibrations of the glycosidic C─O bond	-	-	-	-	1132.48	87.04	1154	84.98
	1095	88.91	1079.39	90.08	-	-	-	-

ν (C-O-C) stretches from carbohydrates								
Sulfate groups as S = O, C−O−S and ν(C-S-C) of sulphated polysaccharides [55] (Trabelsi, M'sakni, Ouada, Bacha, & Roudesli, 2009)	1046.78	86.06	1044.74	89.10	-	-	-	-
δ (C-O-H) deformation, ν (C-O-C) stretches of polysaccharides from carbohydrates, ν_s (P=O) symmetric stretches from nucleic acid	1003.93	84.26	1001.93	91.14	991.68	69.28	1018	75.86
P-O-C stretch, Aromatic phosphates	924.35	84.90	-	93.64	-	-	-	-
the bands at 827 cm−1 and 887 cm−1 indicated the coexistence of α and β-configurations of the sugar units [51,52] (Harshal & Lalitha, 2014; Tang et al., 2020)	863.13	97.85	865.17	92.75	869.25	77.49	879	84.58
bending band of N-H and C-N from the amide III band	838.6	86.68	832.52	92.75	822.32	78.56	-	-
Vibration of C-O bonds for an epoxy function	706.01	84.45	703.97	90.25	-	-	-	-
C-Cl stretching, Aliphatic Chloro compound	644.8	81.42	-	89.99	-	-	-	-
C-Br stretching, Aliphatic bromo compounds	614.19	80.17	-	87.40	-	-	-	-
